# Beyond clinical trials: Evolutionary and epidemiological considerations for development of a universal influenza vaccine

**DOI:** 10.1371/journal.ppat.1008583

**Published:** 2020-09-24

**Authors:** Cécile Viboud, Katelyn Gostic, Martha I. Nelson, Graeme E. Price, Amanda Perofsky, Kaiyuan Sun, Nídia Sequeira Trovão, Benjamin J. Cowling, Suzanne L. Epstein, David J. Spiro

**Affiliations:** 1 Division of International Epidemiology and Population Studies, Fogarty International Center, National Institutes of Health, Bethesda, Maryland, United States; 2 Dept. of Ecology and Evolutionary Biology, University of California, Los Angeles, Los Angeles, California, United States; 3 Dept. of Ecology and Evolution, University of Chicago, Chicago, Illinois, United States; 4 Center for Biologics Evaluation and Research, Food and Drug Administration, Silver Spring, Maryland, United States; 5 World Health Organization Collaborating Centre for Infectious Disease Epidemiology and Control, School of Public Health, The University of Hong Kong, Hong Kong Special Administrative Region, China; University of Alberta, CANADA

## Abstract

The prospect of universal influenza vaccines is generating much interest and research at the intersection of immunology, epidemiology, and viral evolution. While the current focus is on developing a vaccine that elicits a broadly cross-reactive immune response in clinical trials, there are important downstream questions about global deployment of a universal influenza vaccine that should be explored to minimize unintended consequences and maximize benefits. Here, we review and synthesize the questions most relevant to predicting the population benefits of universal influenza vaccines and discuss how existing information could be mined to begin to address these questions. We review three research topics where computational modeling could bring valuable evidence: immune imprinting, viral evolution, and transmission. We address the positive and negative consequences of imprinting, in which early childhood exposure to influenza shapes and limits immune responses to future infections via memory of conserved influenza antigens. However, the mechanisms at play, their effectiveness, breadth of protection, and the ability to “reprogram” already imprinted individuals, remains heavily debated. We describe instances of rapid influenza evolution that illustrate the plasticity of the influenza virus in the face of drug pressure and discuss how novel vaccines could introduce new selective pressures on the evolution of the virus. We examine the possible unintended consequences of broadly protective (but infection-permissive) vaccines on the dynamics of epidemic and pandemic influenza, compared to conventional vaccines that have been shown to provide herd immunity benefits. In conclusion, computational modeling offers a valuable tool to anticipate the benefits of ambitious universal influenza vaccine programs, while balancing the risks from endemic influenza strains and unpredictable pandemic viruses. Moving forward, it will be important to mine the vast amount of data generated in clinical studies of universal influenza vaccines to ensure that the benefits and consequences of these vaccine programs have been carefully modeled and explored.

## Author summary

There is a lot of interest in developing universal influenza vaccines that could elicit broad protection against multiple strains of influenza viruses. This impetus is generating intense basic science research in the field of influenza immunology, in the hope that new vaccine candidates can be identified, reach clinical trials, and deliver robust influenza responses. However, there are important downstream questions about using universal influenza vaccines in large populations that should be considered during the development and implementation of these novel products. Important questions include how early childhood influenza exposure limits immune responses to future influenza infections, how universal influenza vaccines could drive viral evolution, and how these new vaccines could impact the epidemiology of influenza in epidemic and pandemic seasons. Here, we synthesize the questions most relevant to predicting the population impacts of universal influenza vaccines and discuss how existing data could be analyzed to address these questions.

## Introduction

In today’s interconnected world, human influenza A viruses emerge, evolve, and spread globally, eluding host defenses and the best efforts of vaccine manufacturers. A current public health goal is to develop influenza vaccines that provide broad protection against all influenza A viruses capable of infecting humans [1]. These “universal influenza vaccines” would significantly reduce global morbidity and mortality from seasonal influenza epidemics, while also protecting populations against the potential emergence of novel pandemic influenza viruses from animal reservoirs, including swine and poultry. This article explores the potential population-level consequences of universal vaccines on immunity, viral evolution, and transmission and identifies gaps in extant experimental and observational data. Two recent articles [2, 3] offer complementary views on the optimization of universal influenza vaccines and address disease dynamics between and within hosts.

Although influenza B viruses cocirculate with influenza A viruses and cause annual epidemics, our review is limited to influenza A viruses since they are responsible for most of the annual burden of influenza epidemics, have greater evolutionary rates than influenza B viruses, and exert a unique pandemic threat [4]. In consequence, influenza A viruses are a major target for universal influenza vaccine development. We focus on universal vaccines candidates that target the hemagglutinin (HA) stem, a conserved region of the main surface protein of the influenza virus, since this is a relatively new field that is generating questions that are ripe for computational modeling. We also discuss universal vaccines constructs based on highly conserved proteins such as nucleoprotein (NP) and matrix (M), which have inspired prior modeling work [5]. We take a broad view of computational modeling to include quantitative analyses of epidemiological and phylogenetic data and simulations of disease dynamics under various epidemiological and intervention scenarios.

## Universal influenza vaccines and population immunity

**Fig 1:**
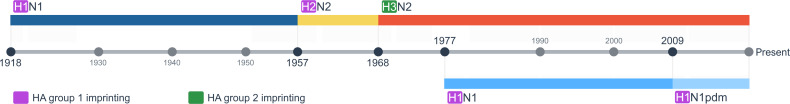
Timeline of influenza A virus circulation in humans from 1918 to present. Colored boxes represent different eras of influenza A subtype circulation and, hence, imprinting of population cohorts by different subtypes. Group-level imprinting is indicated as purple shading for group 1 (H1 and H2 viruses) and in green for group 2 (H3 virus). Cohorts born after 1977 can be imprinted by either group due to cocirculation of H1 and H3 viruses.

Despite decades of research, influenza immunity remains a complex topic about which we only have a limited understanding. Influenza A subtypes are classified based on their surface proteins hemagglutinin (HA) and neuraminidase (NA), with influenza H1N1, H2N2, and H3N2 having circulated in humans since the 1918 pandemic (see Fig 1 for a timeline of antigen circulation). Individuals are exposed to a variety of influenza strains throughout life, while periodic cycling of influenza subtypes and strains shapes the immune response of different birth cohorts. Similarly to many other pathogens, influenza attack rates are highest in immunologically naïve children [6]. Although older individuals experience greater rates of hospitalization and death than the young during seasonal influenza epidemics, older individuals tend to be less affected during pandemics because their immune systems have often already seen similar viruses in childhood. This phenomenon of “senior sparing” was observed in individuals aged 50 years and over during the 2009 H1N1 pandemic, prompting the theory that exposure to related H1N1 viruses in childhood may have conferred long-lasting protective immunity [7]. This epidemiological observation, later confirmed by immunologic studies [8–10], illustrates the existence of lifelong immunity structured by birth year that may alleviate symptoms from influenza infection.

**Fig 2:**
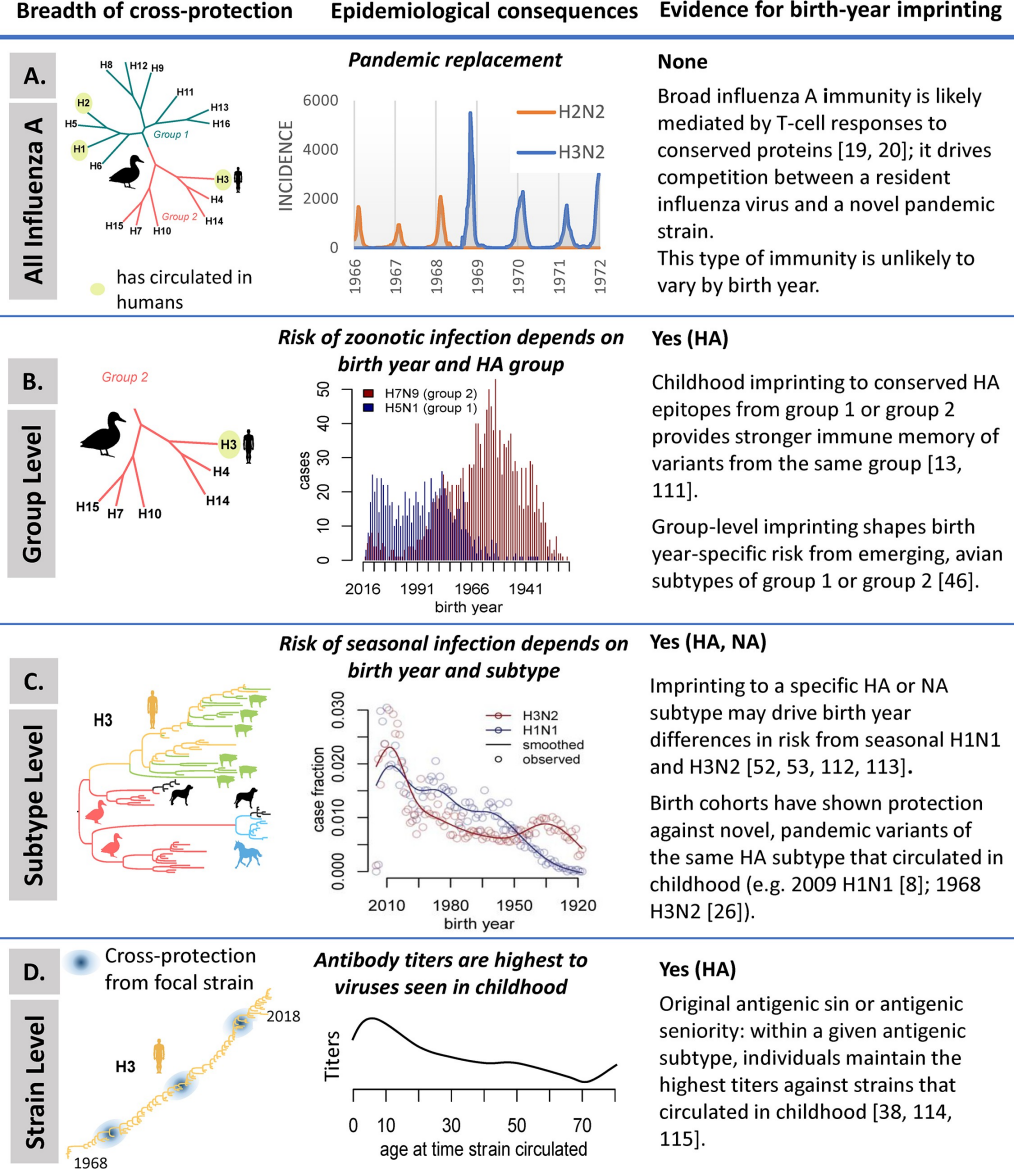
Immune mechanisms involved in influenza protection, with their evolutionary and epidemiological signatures, and evidence for imprinting by birth year. Immune mechanisms are organized by breadth of protection, from broadest to most specific. (A) Protection against all influenza A viruses. (B) Protection at the HA group level. (C) Protection at the subtype level. (D) Strain-specific protection. The strength and duration of protection, and the impact on transmission, remain debated for most of these mechanisms (see also [111–115]).

Broadly protective immunity to influenza could be mediated by a variety of immune responses raised to conserved components of influenza A viruses that circulate in humans (recapitulated in Fig 2A and 2C). It is generally accepted that such broad immune responses do not provide sterilizing immunity and cannot prevent infection. However, they can modulate the course of infection by reducing viral titers, disease severity, duration of infection, and onward transmission, as shown by a variety of animal and human studies (See [5] for an illustration of modeling of such data). Plausible mechanisms of broadly protective immunity include antibody responses to the stem of the HA [11], which appears to be more evolutionarily constrained than other parts of the protein, or antibody responses to stable epitopes on the HA head that can persist for decades or reappear [8, 12, 13]. Further, antibody responses against NA can be broadly protective [14–16] and have been implicated in protection against pandemic viruses [17]. In addition, it has been long suspected from epidemiological data that T-cell memory responses to conserved proteins of the virus (e.g., nucleoprotein NP, matrix M1 and M2e, and polymerase PB1) may also play an important role in pandemic protection [18]. A protective role of T-cell responses is supported by modern immunologic assays in humans [19, 20] and epidemiological studies reporting how influenza T-cell escape variants can spread in populations [21]. In addition, non-neutralizing antibody responses to conserved proteins M2 [22, 23] and NP [24] can contribute to protection.

Replacement of a resident influenza subtype by a new pandemic virus within a year or two of pandemic virus emergence, as occurred during the 1957 and 1968 pandemics, provides strong population-level evidence for broadly protective immunity induced by one subtype and protective against another subtype (Fig 2A). Replacement signals competition between influenza A subtypes in human populations, likely mediated by heterosubtypic cross-immunity. Various heterosubtypic cross-protective immune mechanisms likely act in combination (e.g. B cell or T cell mediated) and their impact on infection, disease, and transmission remains difficult to untangle [10, 17, 18, 25, 26, 27, 28]. As a case in point, following the 2009 influenza pandemic, seasonal H1N1 viruses were replaced by the new H1N1 pandemic virus, likely due to cross-immunity to conserved regions of these relatively distant viruses [11]. Harnessing broadly cross-protective immune mechanisms is critical for the development of universal influenza vaccines, and dissecting their effects over a lifetime of influenza exposures in large populations will help guide the development and implementation of universal influenza vaccines. Below, we review broadly protective immune mechanisms in detail, their putative population profiles, and their expected impact on disease dynamics.

### The HA stem is a proposed target for universal influenza vaccines

The HA stem is a relevant region of the influenza virus for vaccine purposes because it is conserved across different influenza subtypes that infect host species that may be sources of new pandemic variants. To date, 16 antigenically distinct HA subtypes (H1-H16) have been identified in wild birds, the reservoir of influenza viruses. Based upon HA stem sequences, HAs segregate phylogenetically into two major groups: Group 1 includes the human seasonal H1 and avian H5 subtypes, as well as the H2 subtype that circulated in humans during 1957–68, while group 2 includes the human seasonal H3 and avian H7 strains (Fig 2B). Monoclonal antibodies specific to the HA stem and present in the serum of influenza-infected individuals are usually group specific but may not bind all virus strains within a group. Rare antibodies have also been identified that bind to both group 1 and group 2 viruses [29–31].

Designing vaccines that generate strong HA stem-specific responses is difficult in practice because the HA stem is typically immunosubdominant to the HA head [32]. Thus, innovative vaccination strategies based on chimeric HAs or stabilized headless stems have been used to focus the immune response away from the immunodominant head to the less immunogenic stem. These strategies elicit broad cross-protective immunity against a wide range of influenza subtypes and historical strains in animal models, and Phase I clinical trials of such vaccines have been initiated [33–35]. While monoclonal antibodies directed against the HA stem exhibit neutralizing activity in vitro, they also act via mechanisms like antibody-dependent cellular cytotoxicity, which do not block viral entry into cells [36]. Due to these immune mechanisms, and/or imperfect cross-reactivity with circulating stem epitopes, an HA stem-based vaccine may be an infection-permissive or “leaky” vaccine.

### Immunological imprinting

Here, we use the term immunological imprinting to refer to the theory that influenza exposures early in life shape and limit future immune responses to influenza infection; by this definition, imprinting is related to birth year. This concept originated in 1960, when Thomas Francis coined the term”Original Antigenic Sin” (OAS) to describe how the first influenza infection “governs the response to vaccination with other strains” [37] (Fig 2D). The theory of antigenic seniority is a variation on OAS, in which there is a hierarchy of immune responses to viruses encountered during a lifetime, with the first virus generating the highest antibody titers compared to viruses seen later in life [38]. A possible mechanism explaining antigenic seniority is back-boosting, whereby initial influenza responses are amplified upon re-exposure to later influenza variants that share epitopes with the earlier strains [39]. The theories of antigenic sin and seniority have been primarily developed to interpret antibody responses to variable epitopes on the head of the H3 HA [40]. The concepts of imprinting, OAS, antigenic seniority, and back-boosting all relate to the unique consequences of early life exposures. The mechanisms behind immune imprinting and even the existence of OAS are being actively debated [40, 41].

While the theory of OAS has been in the literature for decades, the immunological basis for the phenomenon is poorly understood. The first exposure to influenza in childhood has been shown to generate a very narrow antibody response, with little cross-reactivity to other influenza hemagglutinin subtypes [42]. Subsequent exposure to a related influenza virus that has undergone antigenic drift stimulates a small subset of memory B cell clones that respond to regions of the HA head and/or stem that are cross-reactive with those encountered earlier [13]. Responses to previously seen epitopes can increase year after year as long as strains cross-reactive at that site are circulating (Fig 2D). Early influenza infections can therefore leave a lifelong imprint or “bias” in an individual’s immune repertoire [9], and, in turn, population-level immunity is expected to be biased and structured by birth year. Imprinting could also affect other aspects of influenza immunity, including antibody responses to the NA [43] or T-cell responses [44].

Other types of interference can occur between memory B cells and de novo immune responses that are unrelated to birth year but can cause the immune system to become hyperfocused on familiar epitopes, leaving the host unprepared against a drifted strain [40]. As a case in point, impaired immune responses after routine annual vaccination may arise from repeat exposure to highly similar antigens. This phenomenon has been observed across a broad range of age groups and, hence, is distinct from birth year imprinting [40, 45].

### Computational models can test immunological hypotheses

Support for imprinting can be found in epidemiological analyses of population-level surveillance data, particularly by comparing the risk of influenza infection in birth cohorts imprinted by different influenza histories (Fig 2B and 2C). A long-standing epidemiological puzzle had been the stark contrast in the age mortality profiles of individuals infected with avian-origin H5N1 and H7N9 viruses in Asia. A recent study revisited these data under the imprinting hypothesis, highlighting how the *birth year* profiles of cases aligned with the switch between circulation of group 1 and group 2 HA in 1968 ([46], Fig 2B). Individuals born prior to 1968 are likely imprinted by group 1 HA (H1N1 or H2N2 viruses); these individuals are more susceptible to zoonotic viruses carrying a group 2 HA, such as H7N9. Conversely, younger individuals born after 1968 are more likely to be first exposed to group 2 HAs (H3N2 viruses) and may, therefore, be more susceptible to zoonotic viruses belonging to the other group, including H5N1. These epidemiological observations are consistent with the theory of imprinting at the HA group level. Monoclonal antibodies to the HA stem are generally specific to an HA group (reviewed in [47]), suggesting a role for stem antibodies in the protection against zoonotic influenza strains [46, 48].

While immune imprinting may generate lifelong antibody responses to conserved epitopes and offer partial protection against certain zoonotic strains and pandemics, it may also have detrimental effects during seasonal outbreaks. When a new influenza virus variant emerges through the process of antigenic drift, some individuals may recall memory B cells that target variable epitopes of the HA head of related strains seen earlier in life, but these responses may not be sufficiently well-matched to block infection. For example, individuals who had their first exposure to H1N1 in the period from 1977 to 1986 may have focused their response to a particular epitope of the HA head (K166) that was similar to, and protective against, the 2009 pandemic H1N1 virus. When this region of H1N1 viruses acquired substitutions during the 2013 to 2014 influenza epidemic, these middle-aged cohorts experienced higher disease burden than in prior seasons and responded more poorly to seasonal influenza vaccines [49, 50]. Further, group-level imprinting may not be universally protective against pandemic strains; cohorts imprinted by H2N2 may have experienced increased mortality risk during the 2009 pandemic, relative to surrounding cohorts [51].

### Modeling risk of infection by birth year

Computational models can be useful to test the plausibility of various imprinting mechanisms given a set of surveillance observations (Fig 2C). Testable hypotheses include whether there is evidence of lifelong immune protection at the HA group level or narrower protection at the level of the HA or NA subtype. Another question of interest is whether HA imprinting is stronger for group 1 or group 2 viruses. A number of studies have started to confront these immunologic scenarios by analyzing influenza surveillance data stratified by birth year and virus subtype [52, 53]. The early life influenza exposure of each birth cohort is reconstructed probabilistically, based on information on influenza circulation since 1918. Critical breakpoints in these studies are the pandemic years of 1957, 1968, and 1977, which signal the beginning of new influenza exposure histories for cohorts born in distinct influenza eras. Reconstruction of exposure histories after 1977 is more complicated due to cocirculation of H1N1 and H3N2 viruses since that time (Fig 1). Detailed information on the intensity of H1 and H3 epidemics each year in various locations is important to accurately model exposure histories but difficult to obtain.

Recent analyses of the risk of seasonal influenza infection have illuminated the role of birth year imprinting at the HA and NA subtype levels [52, 53] (Fig 2C). Those primed by H1N1 (born before 1957) experience partial protection against all seasonal influenza H1N1 viruses throughout life but no advantage against H3N2 infections. The converse is true of those primed by H2N2 or H3N2. It is difficult to disentangle from seasonal data whether this type of subtype-level protection originates from priming with HA and NA, or both, as both hypotheses have similar statistical support. However, seasonal influenza data do not support the contribution of immunity at the HA group level. Computational models are particularly useful here as they take into account age-differences in susceptibility and behavioral factors that drive the risk of influenza infection and propensity to seek care and, in turn, affect the data collected by surveillance systems [46, 52–55]. Models of seasonal surveillance data [52, 53] have tested for complex combinations of biological hypotheses such as age-specific risk for finely resolved age groups; birth year risk related to imprinting by HA, NA, or both; antigenic distance from the last season; and demographic age distribution. Existing surveillance data support birth year imprinting by HA and NA, superimposed on age-specific risks and demography [52, 53].

### Modeling immune responses by birth year

In addition to modeling of population-level surveillance data, mechanistic models can guide interpretation of immunologic data collected from prospective cohort studies or cross-sectional surveys [38, 54, 56]. Existing research has primarily focused on interpretation of serologic markers, through the lens of hemagglutination inhibition titers, to determine cross-reactivity between antibody responses to different influenza strains (see profile of HA antibody titers throughout a lifetime of exposure in Fig 2D). In the future, modeling approaches could be applied to a broader range of biologic markers that are becoming available to fully capture the specificity of the immune profile of different birth cohorts. Analysis of somatic hypermutation in cohorts of varying birth years and ages could help define the changing effect of immune imprinting over the human life span [57]. Birth year analyses of T-cell responses would provide a better sense of the role of cellular immunity in imprinting. Mathematical models are useful to interpret immunological markers because they can incorporate measurement errors that affect biological assays [58, 59]. For instance, the hemagglutination inhibition test is prone to measurement errors and repeatability issues, and to be conservative, a 4-fold rise over baseline is typically defined as evidence of infection. Because of this strict definition, a fraction of true infections may be missed. In a Vietnamese cohort study, a “data-augmentation” approach was applied to reconstruct the distribution of unobserved infections, given a set of hemagglutinin inhibition titers in a large population and repeat serology in a subset of individuals [58]. The analysis showed that a fraction of individuals with a weak (2-fold) rise in titers could not be explained by measurement error alone and that applying a strict 4-fold criteria would underestimate attack rates [58]. Modeling of serologic data is an active area of influenza modeling research; it is expected to contribute to our understanding of the build-up of population immunity over a lifetime of exposure.

### Can vaccines exploit imprinting?

School-age children are considered high-transmitter groups for influenza, due to high susceptibility to influenza, increased contacts, and long duration of viral shedding [60, 61]. Hence, large-scale pediatric immunization programs have the potential to provide herd immunity benefits to all age groups, including seniors who fail to mount a robust immune response to vaccination [62–64]. Given the importance of children for vaccination programs, a key area for computational modeling in the coming years will be to extract signals of imprinting in surveillance data to determine whether imprinting primarily results from a child’s first, or first few, natural infection(s). A related question is whether the negative consequences of imprinting, if any, can be overcome later in life by subsequent infections or vaccinations. Longitudinal birth cohort studies will be particularly informative to compare the development of immune responses between children in high and low vaccination settings [65]. Another important question is whether development of broadly cross-protective immunity is impaired in vaccinated infants, compared to unvaccinated infants whose first exposure to influenza is via natural infection. Findings might in turn lead to strategies that promote development of a long-lasting broadly protective immunity among infants and children through universal influenza vaccines. However, the difficulty of such analyses should not be underestimated; large, high-quality data sets collected over several decades would be necessary to detect what may be small differences in the strength of imprinting protection between cohorts of vaccinated and unvaccinated children. On the other hand, influenza attack rates are high in children, which may help identify a signal of protection more quickly than in vaccine studies focused on adults. Although rapid age de-escalation of Phase II and III studies would increase power to detect protection, there is a trade-off with the need for careful monitoring of safety signals in these vulnerable populations.

In the United States, it is recommended that infants be immunized at six months of age with tri- or quadrivalent inactivated vaccines containing both group 1 and group 2 strains. It remains unclear whether a first exposure to influenza in the form of conventional vaccines could influence immune imprinting and, in turn, responses to novel pandemic strains, differently than priming by natural infection. Ideally, universal vaccines could imprint naïve children with broad antigenic diversity; exposure to a full spectrum of antigens including the HA stem and conserved proteins such as M2 and NP could be beneficial. So far, it is well-accepted that the strength of immune memory conferred by conventional vaccines is weaker than that of natural infection [16, 66].

If studies determine that OAS cannot be overcome by vaccination or later life influenza exposures, a universal influenza strategy that establishes broadly protective influenza immunity in children might not be effective for the elderly, who are vulnerable to influenza. The ability of the elderly to adjust to rapidly drifting influenza strains declines with age, potentially due to decreased somatic hypermutation [57], reduced B cell clonal diversity [67], or other aspects of immune senescence. Repeat annual immunization with conventional vaccines may narrow immune responses in the elderly even further [56]. While Phase I trials of inactivated adjuvanted vaccines targeting the H1 stem have shown promising immunogenicity results in adults [34], data on the strength and duration of protection are not available yet. Meanwhile, T-cell vaccines have been shown to elicit responses in healthy adults who have experienced multiple influenza exposures [68] and provide protection against challenge virus [69]. Efforts directed toward stimulating T-cells with the influenza internal proteins NP and M1, M2, using multiple peptides, viral vectors, and DNA constructs are among the most advanced universal influenza vaccine programs [68–71]. Phase I and Phase II clinical trials have shown promise in eliciting CD4 and CD8 T-cell responses, reducing viral shedding and symptoms, and producing broadly cross-reactive responses. However, a Phase IIB clinical trial of an NP+M1 vaccine was recently discontinued for failing to meet a predefined endpoint of reduction in incidence and viral shedding [72]. It is plausible that an optimal universal vaccine will require multiple antigens (e.g., HA stem, NA, NP, M2, and/or M1) to elicit B cell and T-cell mediated immunity and provide long-lasting protection across birth cohorts or age groups that have already been imprinted. Moreover, different combinations of antigens inducing different breadth, duration, and strength of protection might be developed as products intended for different age cohorts.

Taken together, these examples illustrate the complexity of influenza immune responses and how computational modeling could be useful to disentangle competing biological mechanisms, project the fate of different birth cohorts responding to seasonal and universal influenza vaccination, and integrate important aspects of influenza epidemiology. High-quality data will be important to inform such models. Modeling will also be useful to synthesize different lines of evidence from a diverse set of experimental and observational studies that have been launched to aid universal influenza vaccine development.

## Universal influenza vaccines and viral evolution

The evolution of influenza A viruses is notoriously difficult to predict. Not only does the virus evolve through rapid mutation of surface HA and NA proteins to evade antibody detection, but entire segments of the genome can be reshuffled through reassortment between different viruses coinfecting the same cell. The evolution of the HA protein follows a distinct ladder-like pattern in humans, driven by continual antibody-induced selection that results in global sweeps of new variants every three to five years. Manufacturing effective seasonal vaccines requires biannual predictions of the next year’s dominant strain, a process that has been informed in recent years by the increased availability of genetic sequence data, including from understudied tropical and subtropical regions, and the development of new visualization tools and predictive methods [73]. Recently, the H3N2 tree has branched out into multiple cocirculating clades, further complicating efforts to predict the next season’s dominant strain. It remains difficult to observe immune-driven selection within a single host [74], and it has been suggested that new variants may be more likely to emerge in people with prolonged influenza infections (for instance, in immune-compromised hosts) [75]. Intriguingly, de novo mutations that emerged in a small sample of immune-compromised hosts were later observed to dominate globally [75]. Between hosts, viral evolution is promoted by longer serial chains of transmission and is amplified by host heterogeneity along the chains [76].

### Evolutionary pressure from universal influenza vaccines

The antigenic evolution of the virus in humans is thought to be driven by host immune pressure resulting from natural infection, with little evidence for vaccines affecting the long-term epidemiology or evolution of the virus [77, 78]. However, it is important to explore the potential for a broadly protective vaccine to modulate influenza virus evolution, since the vaccine could redirect the immune response towards proteins not targeted by natural immunity, potentially introducing new selection pressures. Selection could also be strengthened during a pandemic, with high vaccine utilization on a global scale. It has been theorized that a broadly protective influenza vaccine, targeting conserved proteins NP and M2 and deployed at sufficiently high levels, could actually slow the evolutionary rate of the virus by substantially reducing the number of infected individuals [5]. Vaccination is generally expected to decrease the intensity and duration of shedding and shorten chains of influenza infection, potentially decreasing the risk of escape variants [79]. At the same time, a widely deployed universal influenza vaccine could select for escape mutants in sites that were not previously under immune pressure, especially if this vaccine does not provide sterilizing immunity (infection-permissive or “leaky” vaccines). The risk of escape mutants from a leaky universal influenza vaccine has been considered low because existing vaccine candidates tend to target conserved regions of the influenza virus, which show limited genetic variability in circulating strains. However, restrictions on influenza evolution observed in experimental settings are not always borne out in the real world. For example, the influenza A virus has evolved resistance to antiviral drugs in genetic regions where experimental evidence had suggested that resistance mutations would impair viral fitness [80].

### Influenza viruses evolve resistance to antiviral drugs via multiple pathways

To understand the plasticity of the influenza virus, it is worth exploring two examples involving resistance to antiviral drugs. First, in 2005, H3N2 viruses that were resistant to the adamantane class of antivirals rapidly reached fixation globally, leading to the entire discontinuation of this class of drugs [81]. Phylogenetic analysis revealed that resistance was conferred by a single S31N amino acid substitution in the M2 protein. The S31N substitution appears to have originated in Asia, where cheap, over-the-counter adamantane drugs were anecdotally more widely used than in Europe and North America [82, 83]. The global dissemination of the resistant viruses was accelerated by a genomic reassortment event, in which the resistant M2 gene hitchhiked with an antigenically novel HA that was strongly selected for [82, 83].

In a second notable case study, in 2007, seasonal H1N1 viruses emerged with a NA gene carrying a single amino acid substitution (H274Y) that conferred resistance to a newer antiviral, oseltamivir. Again, the drug resistant viruses rapidly disseminated globally, an observation that was particularly alarming because (1) oseltamivir is a first-line drug and widely stockpiled for use in a pandemic and (2) oseltamivir-resistant viruses had low observed fitness in experimental studies [80]. The spatial and evolutionary origins of the oseltamivir-resistant mutation have not been well characterized ecologically. However, experimental studies have elucidated the role of “permissive” mutations in the NA that predate the H274Y change and have reconfigured the protein to make it capable of acquiring resistance mutations without loss of fitness [80]. This study of oseltamivir resistance demonstrates the importance of genetic context and the range of evolutionary pathways by which influenza viruses circumvent functional constraints. Epistatic interactions and compensatory mutations remain an understudied but highly relevant area of evolutionary research. In the same vein, cytotoxic T lymphocyte (CTL)-escape variants are thought to be limited by strong functional constraints and the high polymorphism of human leukocyte antigens (HLA) [84]. However, a CTL-escape mutation located in a conserved region of the internal NP protein spread rapidly during the 1993 to 1994 epidemic, providing further demonstration of the evolutionary plasticity of influenza A viruses and our inability to predict evolutionary constraints from available data [21].

### Evolution in the head versus stem of the HA

Experimental data indicate that the HA head region has a higher mutational tolerance than the HA stem [29, 85, 86], but in vitro studies have demonstrated that escape mutants from broadly neutralizing antibodies can still occur in the stem [85, 87, 88]. Stem escape mutants arise at a lower frequency than head escape mutants [29], especially under polyclonal immune response. However, stem escape mutants can replicate and some variants do not seem to have impaired replication fitness in vitro and retain virulence in mice [89]. It is possible that the high conservation of the HA stem in nature arises from the region not being targeted by host antibodies, due to the strong immunodominance of the HA head. If this is the case, by retargeting immune responses towards the stem, vaccination could in theory induce higher selection pressure on the stem. Systematic in vitro exploration of the viability of mutations in the HA stem is useful [85, 89, 90], but genetic context is critical. Large-scale bioinformatic studies of circulating virus variants may inform how specific mutations would fare on a population level, or in a different viral genetic background.

The capacity of influenza viruses to evolve resistance to antiviral drugs does not necessarily translate to vaccines. In general, resistance to drugs evolves far more rapidly than to vaccines, for multiple reasons [91]. Drugs are delivered therapeutically, when viral diversity and pathogen populations within a host are high. In contrast, prophylactic administration of vaccines typically prevents or limits infection so that the potential to generate new virus variants is lower. Additionally, drugs tend to target a single biological pathway, and their effects can often be diminished by a single mutation. In contrast, vaccines induce polyclonal immune responses that target multiple antigens and are dependent on host cofactors (e.g., HLAs) that are intrinsically diverse, limiting viral escape at the population level. Therefore, resistance is less likely to emerge in influenza vaccines that prevent or reduce transmission and more likely to emerge in vaccines that narrowly target a small number of epitopes (for example, peptide-based vaccines). This is true whether targeting the HA stem or internal proteins and regardless of whether the targets are thought to be functionally constrained. As a result, the optimal strategy could be to engineer vaccine cocktails targeting multiple immunologic sites, such as MP + NP, or MP + NP + NA + HA stem. Large-scale deep-sequencing analysis of intrahost evolution and transmission events in prospective and household transmission studies across the globe may also be useful to understand how influenza escape mutants arise in individuals and propagate in large populations.

## Impact of universal vaccines on transmission

### Leaky vaccines

In contrast to conventional vaccines, some universal vaccines will likely permit a limited amount of infection and may not block onward transmission. A better understanding of how transmission will be reduced by vaccination is important to anticipate the population-level benefits of universal influenza vaccines and the potential for escape variants [5]. A reduction in transmission will affect influenza dynamics in three ways: (1) Vaccination will decrease circulation of influenza in the community and, hence, reduce the probability of infection in unvaccinated individuals (the so-called herd immunity effect). (2) By reducing opportunities for influenza infection, vaccination may generate pockets of unprotected individuals who are more susceptible to new influenza strains than if they had been naturally infected. This may be particularly important in pandemic seasons [5]. (3) Additionally, as previously noted, a leaky vaccine allowing for viral shedding and onward transmission could promote viral evolution away from the immune sites targeted by the vaccine or other antigenic regions of the virus. The interplay of these different mechanisms will determine the net population benefits and evolutionary consequences of a new vaccine [5]. Because novel influenza variants emerge and circulate globally, increased vaccine pressure in a highly vaccinated population could potentially have trickle down effects in other areas of the world that do not have yet access to the vaccine.

### Correlates of transmission

Estimating the impact of universal influenza vaccines on transmission is important but difficult in practice. One would have to design randomized controlled trials to study the reduction in risk of transmission from vaccinated to unvaccinated individuals. Such trials would be prohibitively expensive due to large sample size, and it is unlikely they would be conducted in early stages of vaccine development or during licensure.

In the absence of direct information on transmission, identification of correlates would be useful, especially if one could rely on variables that are more easily monitored, such as virus shedding or clinical symptoms. Model projections of the population benefits of T-cell based vaccines have used viral shedding data in ferrets vaccinated with NP+M2 recombinant adenovirus vaccines to calibrate transmission effects [5]. Further experimental data support the transmission benefits of vaccination in mice, for recombinant adenovirus vaccines expressing NP+M2 [92, 93] and adjuvanted split virus vaccines [94].

Additional data can be gleaned from household studies, which provide a well-controlled environment to study how transmissibility is affected by the intensity and duration of viral shedding. Difficulties here relate to the putative nonlinear relationship between transmission risk and shedding [95, 96], the different assays used to measure shedding, whether infectious viral titers are quantified (preferably by plaque assay or TCID50, rather than quantitative RT-PCR), and how frequently measurements are taken.

A related question is how much transmission occurs, if any, from asymptomatic or pauci-symptomatic individuals. Ferret experiments indicate that transmission can occur prior to symptom development [97]. In mouse models, transmission was reduced in recipients of a leaky vaccine that renders them asymptomatic or pauci-symptomatic when challenged [92, 93]. Household studies have shown decreased shedding in pauci- and asymptomatic individuals infected with influenza, relative to symptomatic individuals; however, the potential for onwards transmission remains unclear [98]. In humans, the contribution of asymptomatic and pauci-symptomatic transmission could be modulated by the behavior and contacts of individuals with few or no symptoms. A potentially detrimental effect of a vaccine that considerably reduces disease severity might be to increase influenza transmission outside of the home due to increased contacts and mobility, relative to more severely ill individuals. If the transmission reduction conferred by a broadly protective vaccine was compensated by an increase in contacts in those who shed the virus, then broadly protective vaccination programs could result in larger epidemics than well-matched conventional vaccines. This hypothetical issue should be weighed in against the imperfect match of conventional vaccines in seasonal influenza situations and the lack of rapidly available conventional vaccines in pandemic situations. Mathematical models could highlight the particular conditions under which the unintended consequences of universal influenza vaccination could occur and assess the trade-offs against existing formulations [99]. Related work in the context of respiratory syncytial virus illustrates how different characteristics of candidate vaccines may affect disease dynamics positively or negatively [100].

While more work needs to be done to improve our understanding of the relationship between shedding and transmission, there is even less understanding of how symptom severity may affect transmission. Ultimately, predictive models of transmission based on symptoms would open the door to using data routinely collected during vaccine trials.

### Linking animal transmission studies to epidemiological data

Animal models have been used to study influenza transmission potential since the seminal work of Andrewes and Glover in the 1940s and Kilbourne and Schulman in the 1960s [101, 102]. More recent work suggests a correlation between secondary attack rates in households and risk of influenza transmission by respiratory droplets in ferrets, which is auspicious for predictive models based on animal experiments [103]. The mapping between household transmission and experimental data is weaker, however, when direct contact between donor and sentinel ferrets is allowed [103]. Whether there is a relationship between transmission in humans and other influenza models (guinea pigs or mice) remains to be seen. These experiments are costly, and a variety of viruses need to be tested to validate the predictive ability of animal models. A large range of human-to-human transmission potential has been reported for influenza A viruses, from zoonotic viruses that transmit poorly between humans to endemic viruses that generate large epidemics (reproduction number R~0.2 for H5N1 virus to R~3.0 for the 1918 pandemic H1N1 virus [104, 105]). Whether animal models can recapitulate such a broad range of transmissibility remains unclear.

A further barrier is the typically small sample size and lack of standardization of animal transmission experiments, which are underpowered for accurate estimation of transmission risk [106]. Ideally, an initial investment in a large set of experiments could help define a robust relationship between transmission in animals and humans. Smaller and less costly animal studies could then be conducted more routinely as new vaccine candidates become available.

### Quantifying the herd immunity effects of conventional vaccines

Herd immunity is an important consideration for any vaccination program, and there is much to learn here in the context of conventional influenza vaccines that have been used for decades. While these vaccines are regarded as suboptimal with respect to direct protection, their indirect transmission benefits also remain debated. The Japanese experience with mass vaccination of schoolchildren in the 1950 to 1970s, along with modeling studies, have pointed at the herd immunity benefits that would be expected from large-scale influenza immunization efforts [63, 78, 107]. Randomized controlled trials in smaller communities have shown how targeting school-aged children for vaccination can reduce influenza transmission [64, 108]. A large-scale pediatric vaccination program initiated 5 years ago in the United Kingdom generated much enthusiasm but has reported mixed-effects on disease impact [109]. Routine influenza immunization programs have become well-established in high-income countries, and new programs are being rolled out in middle-income settings. The time is ripe for modeling of surveillance data in different geographic contexts to estimate and, possibly, reconcile the transmission benefits of conventional vaccines [62, 78]. Analyses need to control for year-to-year variations in the intensity of epidemics and vaccine effectiveness; comparison of otherwise similar regions with high and low vaccine coverage can be particularly useful here [110]. Another promising avenue includes modeling of detailed individual-level data on the transmission of influenza outbreaks in closed settings, such as military camps, households, or college dormitories, where a fraction of the population is vaccinated.

## Conclusion

The field of universal influenza vaccines is rapidly expanding and generating a vast amount of immunological, virologic, and clinical information, along with important questions on how to optimize these vaccines to maximize protection and minimize harm. Computational modeling can help synthesize these new data streams and contribute to the identification or confirmation of the mechanisms driving immune imprinting on a population level. In parallel, evolutionary studies need to clarify how selection pressures operate at the individual and population levels and drive the global emergence of new strains due to pressure from natural infection, vaccination, or antiviral drugs. Further research is needed to clarify the host and immunologic conditions that increase or decrease the rate of viral evolution and quantify the evolutionary constraints on the different regions of the virus. Combined with data from transmission studies in humans and animals, this information can be used to project the population benefits of universal influenza vaccines. The push towards universal influenza vaccines may generate a complex vaccine landscape with co-existence of different formulations targeting different antigens in different birth and age cohorts. Computational modeling offers a valuable tool to anticipate the benefits of such ambitious programs, while balancing the risks from endemic influenza strains and unpredictable pandemic viruses. Moving forward, it will be important to mine the vast amount of data generated in clinical studies to ensure that the risk-benefits of these vaccine programs have been carefully assessed.
